# Thymic epithelial tumors: what’s next?

**DOI:** 10.1007/s10555-026-10384-5

**Published:** 2026-10-14

**Authors:** Li Zhang, Feiyue Zhang, Mahmoud Ismail, Gaofeng Li, Jens-C. Rueckert

**Affiliations:** 1 Department of Surgery, Competence Center of Thoracic Surgery, Charité-Universitätsmedizin Berlin, Corporate Member of Freie Universität Berlin and Humboldt-Universität zu Berlin, Berlin, Germany; 2 Department of Thoracic Surgery, Klinikum Ernst Von Bergmann, Potsdam, Germany; 3 Department of Oncology, The Sixth Affiliated Hospital of Kunming Medical University, Yuxi, China; 4 Department of Thoracic Surgery, Yunnan Cancer Center, the Third Affiliated Hospital of Kunming Medical University, Kunming, China

**Keywords:** Thymic epithelial tumors, Tumor immunology, Immune checkpoint inhibitors, Molecular profiling, Targeted therapy

## Abstract

Thymic epithelial tumors (TETs) are rare malignancies arising in an organ central to T cell development and immune tolerance, creating a distinctive interface between tumor biology and immunotherapy. Complete surgical resection remains the mainstay for resectable disease, whereas systemic treatment options for unresectable, recurrent, and metastatic TETs remain limited. The unique immunobiology of thymoma, particularly impaired central immune tolerance, may account for the unusual balance between antitumor activity and severe immune-related adverse events observed with immune checkpoint blockade. Recent molecular and immune profiling has begun to reveal biologically distinct TET subtypes and potential therapeutic vulnerabilities, including alterations in cell-cycle, PI3K/AKT/mTOR, RTK/RAS/MAPK, and DNA damage-response pathways, as well as epigenetic and immune regulatory mechanisms. Emerging strategies, including antibody–drug conjugates and biomarker-informed targeted and immune therapies, may expand systemic treatment options for advanced disease. This review examines these developments in the context of the distinctive biology of the thymus and highlights the key unanswered questions that may shape the next generation of TET research and treatment.

## Introduction

Thymic epithelial tumors (TETs) are the most common primary neoplasms of the anterior mediastinum, arising from thymic epithelial cells and encompassing thymomas, thymic carcinomas, and thymic neuroendocrine tumors [1]. According to the WHO classification, TETs are categorized into thymomas (types A, AB, B1, B2, and B3), thymic carcinoma and TNETs, with aggressiveness increasing progressively along this histologic spectrum. Approximately 30% of patients with thymoma present with concurrent myasthenia gravis [2, 3]. Treatment strategies, including surgery alone or combined with neoadjuvant/adjuvant therapies, are determined according to tumor stage and histological subtype, with surgery being the preferred approach for resectable thymoma [4]. Moreover, precision medicine strategies based on comprehensive genomic profiling are being progressively implemented in clinical practice, making precise targeted therapy and immunotherapy emerging options for patients with advanced or metastatic TETs [5]. In this review, we synthesize current evidence on the molecular subtyping of TETs, emerging targeted opportunities, and the unique immunotherapy paradox that distinguishes these tumors from other solid malignancies. We first outline the evolving role of surgery within a precision therapy paradigm, then examine the molecular landscape that underpins subtype-specific therapeutic strategies, and finally critically appraise the clinical evidence for immunotherapy and targeted agents across the TET spectrum.

## Early‑stage TETs: refining resection

Surgery remains the cornerstone of treatment for resectable thymoma, with R0 resection the strongest predictor of long-term survival [6]. Surgical management has progressively shifted from open surgery to minimally invasive approaches. Video-assisted thoracic surgery and robotic-assisted thoracic surgery have increasingly replaced median sternotomy for selected early stage TETs, with comparable R0 resection, recurrence, and survival outcomes, while reducing hospital stay and improving cosmetic results [7–9]. Robotic-assisted thymectomy has further expanded the indications and can be safely applied to selected large thymomas while maintaining oncologic radicality [10, 11].

Complete thymectomy with en-bloc tumor resection has long been the standard surgical treatment for TETs [12, 13]. In patients with thymoma-associated MG, extended thymectomy is associated with superior neurological improvement and lower recurrence rates [14, 15]. However, some studies have suggested that limited thymectomy (thymomectomy alone) would offer improved perioperative outcomes including shorter operative time, less blood loss, fewer complications, and shorter hospital stay while achieving comparable overall survival and recurrence to total thymectomy among patients with early-stage non-MG thymoma [16–20].

Beyond oncologic control, increasing attention has focused on the immunologic consequences of thymic resection. A retrospective study suggests that thymectomy might increase long-term risks of all-cause mortality, malignancy, infection, and autoimmune disease [21]. Conversely, total thymectomy was found to confer superior recurrence-free survival without added perioperative morbidity than thymomectomy alone [22]. In adult patients with localized or small thymomas, thymectomy was not observed to correlate with heightened 5-year mortality or an elevated risk of cancer-related death [23]. Given that the clinical benefits of thymectomy substantially outweigh its potential risks in patients with confirmed thymoma [24], whether limited thymomectomy represents the optimal strategy for selected patients with early stage non-MG thymoma warrants prospective randomized validation.

### Locally advanced-stage TETs: multimodality integration

For locally advanced-stage thymic epithelial tumors, multimodality therapy centered on chemotherapy remains guideline-recommended (Table 1), yet surgical resection, whether upfront or following induction therapy is consistently associated with improved overall survival [25].

**Table 1:** Neoadjuvant Clinical Trials In Thymic Epithelial Tumors

Therapeutic strategy	Trial identifier	Disease setting	Key therapeutic concept	Primary endpoint	Phase
Neoadjuvant treatment intensification	NCT07463313	Potentially resectable locally advanced TETs	Six *vs.* three cycles of neoadjuvant chemotherapy	Event-free survival (EFS)	Phase II/III
Induction chemotherapy optimization	NCT05816694	Thymoma	Nab-paclitaxel–based regimen *vs.* CEP chemotherapy	Overall response rate (ORR)	Phase II
Neoadjuvant radiotherapy	NCT06924619	Locally advanced unresectable thymoma	Radiation-induced downstaging to improve resectability	R0 resection rate	Phase II
Neoadjuvant immunoradiotherapy	NCT06019468	Locally advanced thymic epithelial tumors	Envolizumab plus radiotherapy prior to surgery	Objective response rate (ORR)	Phase II
Neoadjuvant chemoimmunotherapy	NCT03858582	Unresectable TETs (Masaoka stage III/IVA)	Pembrolizumab plus docetaxel/cisplatin as induction therapy	Major pathologic response (MPR)	Phase II
Neoadjuvant PD-1–based therapy	NCT04667793 (ChestMate-001)	Locally advanced thymic epithelial tumors	Toripalimab plus chemotherapy prior to surgery	Frequency of severe adverse events	Phase II

Neoadjuvant chemotherapy has long been integral to multidisciplinary management of locally advanced TETs, and perioperative pembrolizumab has shown potential to improve pathological response, R0 resection rates, and disease-free survival in stage III–IV disease [26, 27]. Complex procedures including superior vena cava resection and vascular reconstruction now enable radical resection in selected stage III–IVa tumors with mediastinal vascular invasion [28–30], while pleural debulking or extrapleural pneumonectomy may offer benefit in carefully selected stage IVa patients with pleural dissemination [31, 32]. Complete resection in selected stage IVb thymoma may prolong survival [33], and even maximal debulking for unresectable locally advanced disease is now considered within individualized multidisciplinary strategies [34, 35].

Postoperatively, adjuvant radiotherapy improves overall survival in stage II–III thymoma [36, 37], confers survival benefit in advanced thymic carcinoma [38, 39] and adjuvant chemotherapy may offer additional benefit in selected high-risk patients [40, 41]. The perioperative treatment landscape is also evolving through clinical evaluation of systemic therapies, including immune checkpoint inhibitors and molecularly targeted agents.

### Advanced TETs: systemic therapy in the immunotherapy era

The systemic treatment landscape of advanced TETs is being reshaped by diverse therapeutic modalities. PD-1/PD-L1-directed immune checkpoint inhibition (ICI) has demonstrated clinical activity (Table 2), but patients with thymoma face substantially higher risks of severe immune-related adverse events than patients with thymic carcinoma [42]. This efficacy–toxicity paradox is starkest when comparing histologic subtypes: pembrolizumab achieves higher responses in thymoma than thymic carcinoma, but at the cost of a nearly five-fold higher rate of severe irAEs (71.4% vs. 15.4%), with life-threatening myocarditis and myasthenia gravis exacerbations occurring exclusively in thymoma [43, 44]. Thymic carcinoma appears more responsive to ICIs, whereas immunotherapy in thymoma demands exceptionally cautious risk–benefit assessment [45]. These findings should not be generalized to all immune checkpoint strategies, because PD-1/PD-L1 blockade, CTLA-4-containing regimens, and combination approaches may differ in both efficacy and toxicity. Navigating this expanding but histologically stratified therapeutic armamentarium requires biomarker-driven patient selection, rigorous toxicity surveillance. Understanding this clinical paradox requires consideration of the unique immunobiology of thymoma.

**Table 2:** Key clinical trials of systemic therapy in advanced thymic tumor

Trial (phase)	Regimen	Population	*N*	ORR (95% CI)	DCR (95% CI)	mPFS (95% CI)	mOS (95% CI)	Grade ≥ 3 AEs	Ref
**First-line**									
RELEVENT (II)	Ramucirumab + Carbo + Paclitaxel → Ramucirumab maint	Advanced TC	35	80.0% (63.1–91.6) [INV]; 57.6% (39.2–74.5) [CIR]	—	18.1 mo (10.8–52.3)	43.8 mo (31.9–NR)	48.6%	[61]
MARBLE (II)	Atezolizumab + Carbo + Paclitaxel → Atezolizumab maint	Advanced/recurrent TC	48	56% (41–71) [CIR]	—	NR	NR	Neutropenia 56%, leukopenia 33%, febrile neutropenia 23%	[62]
**Second-line or later**									
PECATI (II)	Lenvatinib + Pembrolizumab	Pretreated metastatic B3 thymoma and TC	43	—	—	5-mo PFS 88.4% (90% CI 79.8–96.7)*	NR	37%	[63]
STYLE (II)	Sunitinib	Pretreated advanced/recurrent TC	32	21.7% (90% CI 9.0–40.4)	89.3% (71.8–97.7)	8.8 mo (5.3–11.1)	27.8 mo (13.2–53.2)	51.6%	[64]
STYLE (II)	Sunitinib	Pretreated advanced/recurrent B3 thymoma	12	0% (90% CI 0.0–22.1)	91.7% (61.5–99.8)	7.7 mo (2.4–45.5)	47.9 mo (4.5–NR)	25.0%	[64]
Lenvatinib RWS	Lenvatinib	Pretreated advanced TC (real-world)	87	30% (90% CI 21.3–39.1)	93% (84.6–97.2)	10.2 mo (7.0–13.2)	NR (18.3–NR)	84% dose reduction	[110]
NIVOTHYM (II)	Nivolumab	Pretreated advanced/relapsed B3 thymoma and TC	49	12% (5–25)	63% (48–77)	6.0 mo (3.1–10.4)	21.3 mo (11.6–NE)	57%	[111]
Rajan et al. (Phase I)	Avelumab 10–20 mg/kg Q2W	Relapsed advanced thymoma + TC	8	Thymoma: 2/7 (29%) confirmed PR; 2/7 (29%) unconfirmed PR; TC: 0 PR	5/8 (63%)	NR	NR	All responders (thymoma) developed irAEs; 1 non-responder had irAEs	[42]
Cho et al. (Phase II)	Pembrolizumab 200 mg Q3W	Refractory/relapsed TET	33	Thymoma: 28.6% (8.2–64.1); TC: 19.2% (8.5–37.9)	Thymoma: 100% (2 PR + 5 SD); TC: 73.1% (5 PR + 14 SD)	6.1 mo (both groups)	NR	Thymoma: 71.4% (hepatitis, myocarditis, MG); TC: 15.4%	[43]
Giaccone et al. (Phase II)	Pembrolizumab 200 mg Q3W	Recurrent thymic carcinoma (≥ 1 prior chemo)	40	22.5% (10.8–38.5) [1 CR + 8 PR]	75.0% (1 CR + 8 PR + 21 SD)	NR (median FU 20 mo)	NR	15.0% (myocarditis 5%, transaminitis 13%)	[44]

AE adverse event, Carbo carboplatin, CI confidence interval, CIR central independent review, CR complete response, DCR disease control rate, FU follow-up, INV investigator assessment, irAE immune-related adverse event, maint maintenance, MG myasthenia gravis, mo months, NE not estimable, NR not reported, ORR objective response rate, PR partial response, Q2W every 2 weeks, Q3W every 3 weeks, RWS real-world study, SD stable disease, TC thymic carcinoma, TET thymic epithelial tumor

*5-month PFS rate reported instead of median PFS. —, not available

### The biological basis of the immunotherapy paradox in thymoma

**Fig. 1:**
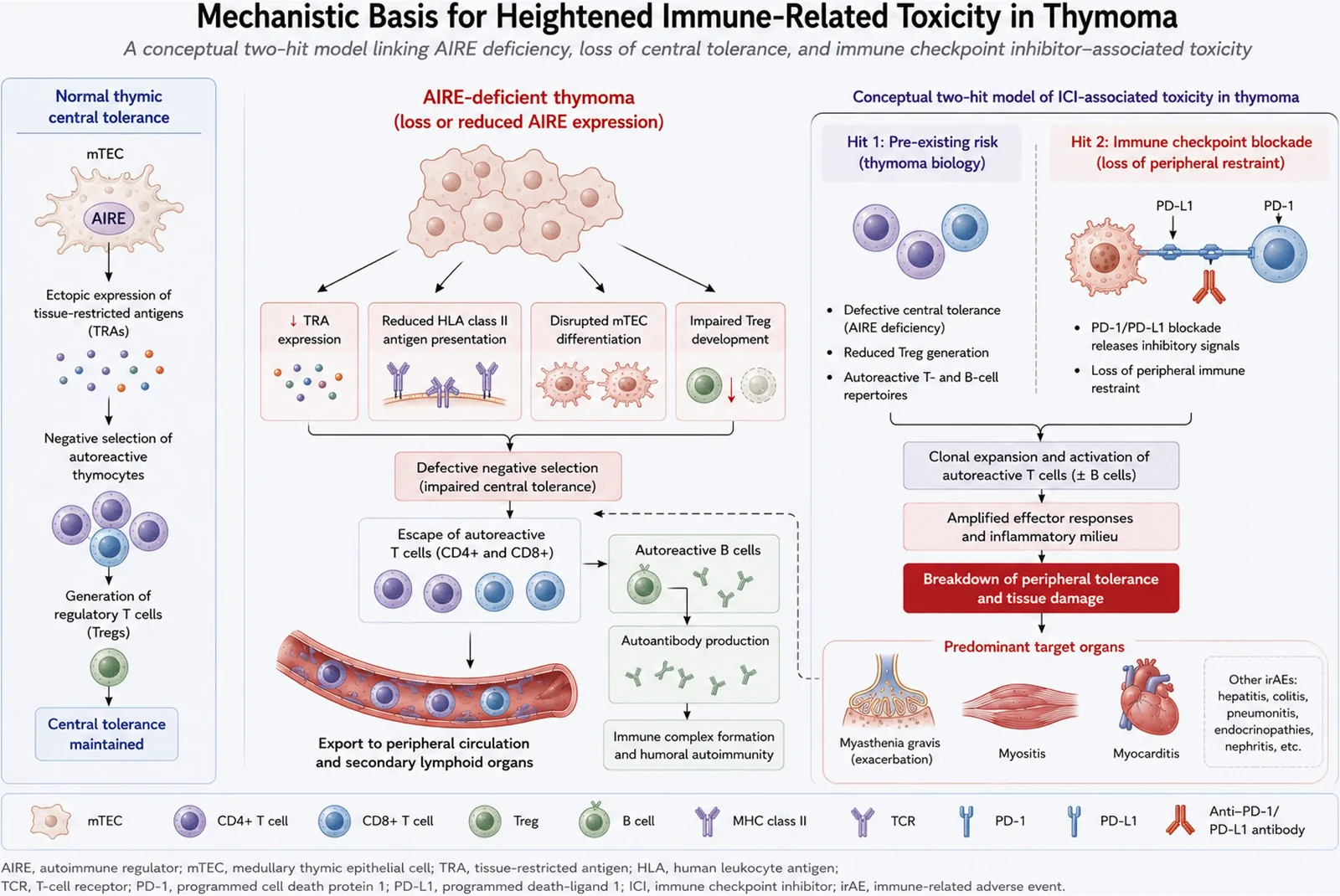
Mechanistic basis for heightened immune-related toxicity in thymoma

The susceptibility of patients with thymoma to severe immune-related adverse events during ICIs therapy may be related to defects in central immune tolerance (Fig. 1). Unlike most solid tumors, thymomas arise from thymic epithelial cells that are essential for establishing central immune tolerance. As the central organ for T cell maturation and maintenance of immune tolerance, the thymus plays a critical role in preserving adaptive immunity [46–48]. In the normal thymus, medullary thymic epithelial cells (mTECs) express the autoimmune regulator (AIRE) transcription factor, which drives the promiscuous expression of tissue-restricted antigens and enables negative selection of self-reactive T cells [49]. However, thymoma exhibits profound disruptions in this process, characterized by reduced AIRE expression, decreased MHC class II expression, and altered tumor architecture that can impair normal T cell selection [50, 51]. Consequently, autoreactive CD4⁺ T cells escape negative selection and are exported to the peripheral circulation, where they coexist with impaired regulatory T cell generation [50]. This baseline state characterized by a circulating pool of autoreactive T cells poised to recognize self-antigens creates a “pre-primed” immune system [52]. When immune checkpoint inhibitors such as anti-PD-1/PD-L1 antibodies are administered, they release the brakes not only on antitumor T cells but also on these preexisting autoreactive clones, precipitating rapid and often severe immune-related adverse events that disproportionately affect muscle and cardiac tissue (including myocarditis, myositis, and myasthenia gravis exacerbations) [52]. A hypothesis proposes a conceptual “two-hit” model in which intrinsic defects in central tolerance represent the first hit, whereas immune checkpoint inhibition constitutes the second hit, together resulting in exaggerated immune activation and a markedly increased risk of severe immune-related adverse events that highlights the need for biomarker-guided patient selection and intensive toxicity monitoring [53]. The model provides a framework for understanding the observed clinical toxicity, but it should be regarded as a conceptual hypothesis rather than an established causal pathway that fully explains all immune-related adverse events in thymoma.

Biomarker development remains critical for optimizing immunotherapy in TETs. PD-L1 expression has emerged as a potential predictive biomarker in thymic carcinoma, and combined evaluation of PD-L1 protein and mRNA expression may further improve prognostic stratification [54, 55]. However, high PD-L1/PD-1 expression is also associated with aggressive histology and poor prognosis, while significant intratumoral heterogeneity poses additional challenges for ICI application in TETs [56–59].

### Emerging therapeutic strategies in thymic epithelial tumors

Beyond immune checkpoint blockade, combination strategies are now reshaping the therapeutic landscape (Table 3) [60]. Multitargeted kinase inhibitors (sunitinib, lenvatinib) and chemoimmunotherapy combinations (ramucirumab or atezolizumab plus carboplatin/paclitaxel) have demonstrated activity in thymic carcinoma [61–64]. In platinum-refractory B3 thymoma and thymic carcinoma, avelumab plus axitinib has shown activity through combined immune checkpoint and antiangiogenic mechanisms [65]. Similarly, ramucirumab added to carboplatin and paclitaxel has shown promising efficacy and safety in previously untreated advanced thymic carcinoma [61].

**Table 3:** Emerging therapeutic strategies trials in thymic epithelial tumors

Therapeutic strategy	Mechanism/category	Representative trial	Disease setting	Primary endpoint
**Immune checkpoint inhibitors**				
Pembrolizumab	PD-1 immune checkpoint blockade	NCT02607631	Platinum-refractory advanced TETs	Objective response rate
Nivolumab	PD-1 immune checkpoint blockade	NCT03134118 (NIVOTHYM)	Pretreated advanced B3 thymoma/TC	6-month PFS rate
Bintrafusp alfa	Dual PD-L1/TGF-β inhibition	NCT04417660	Recurrent TETs	Objective response rate
**Targeted therapies**				
Selinexor	XPO1 inhibition	NCT03466827	Advanced/refractory inoperable TETs	Overall response rate
Rivoceranib	VEGFR-2 inhibition	NCT06200233	Metastatic TETs resistant to first-line therapy	Objective response rate
Ramucirumab + carboplatin/paclitaxel	Antiangiogenic combined chemotherapy	NCT03921671	Relapsed/metastatic thymic carcinoma or B3 thymoma	Best tumor response (CR + PR)
Palbociclib	CDK4/6 inhibition	NCT03219554	Recurrent/metastatic advanced TETs	Progression-free survival
**Antibody–drug conjugates (ADCs)**				
Sacituzumab tirumotecan	TROP2-directed ADC	NCT07343453	Advanced TETs after prior therapy	Objective response rate
Sacituzumab govitecan	TROP2-directed ADC	NCT06248515	Advanced thymoma/thymic carcinoma	Overall response rate
**Other novel biotherapies**				
PT-112	Immunogenic cell death induction	NCT05104736	Recurrent TETs	Overall response rate
Lu-177 DOTATATE (Lutathera)	Peptide receptor radionuclide therapy (PRRT)	NCT06121271	Metastatic somatostatin receptor-positive neuroendocrine tumors	Safety
**Immuno-radiotherapy (Abscopal Strategies)**				
SBRT + rhGM-CSF + peginterferon alfa-2b	Abscopal immunoradiotherapy	NCT04517539	Metastatic TETs	Abscopal effect rate
Radiotherapy + rhGM-CSF	Immune-modulated radiotherapy	NCT05407649	Recurrent/refractory advanced TETs	Abscopal effect rate
**Precision/particle radiotherapy**				
Hypofractionated radiotherapy	Precision radiotherapy	NCT06692062	Masaoka–Koga stage I–III disease	3-year progression-free survival
Hypofractionated RT + chemotherapy + thymosin α1	Combined immunochemoradiotherapy	NCT03663764	Unresectable/recurrent TETs	Progression-free survival
Proton radiotherapy	Cardio-pulmonary sparing RT	NCT06311968	Postoperative R0/R1 TETs	Progression-free survival and toxicities
Heavy-ion radiotherapy	Particle therapy escalation	NCT06311955	Postoperative R2 disease	Progression-free survival and toxicities
Carbon-ion radiotherapy + chemotherapy	High-LET precision radiotherapy	NCT06186726	Inoperable locally advanced TETs	Disease progression-free survival
**Locoregional/intrathoracic chemotherapy**				
HITOC combined with surgery	Locoregional intrathoracic chemotherapy	NCT07383142	Pleural dissemination/recurrence	3-year progression-free survival
Surgery followed by HITOC	Multimodality pleural disease control	NCT05446935	Pleural spread/recurrence	Postoperative hospital stay
HITOC after electrocautery resection	Regional hyperthermic chemotherapy	NCT07328074	Pleural metastasis/recurrence	Event-free survival

ADC antibody–drug conjugate, CR complete response, HITOC hyperthermic intrathoracic chemotherapy, LET linear energy transfer, ORR objective response rate, PD-1 programmed cell death protein 1, PD-L1 programmed death-ligand 1, PFS progression-free survival, PR partial response, PRRT peptide receptor radionuclide therapy, rhGM-CSF recombinant human granulocyte–macrophage colony-stimulating factor, RT radiotherapy, SBRT stereotactic body radiotherapy, TETs thymic epithelial tumors, TGF-β transforming growth factor beta, TROP2 trophoblast cell surface antigen 2, VEGFR-2 vascular endothelial growth factor receptor 2

**Fig. 2:**
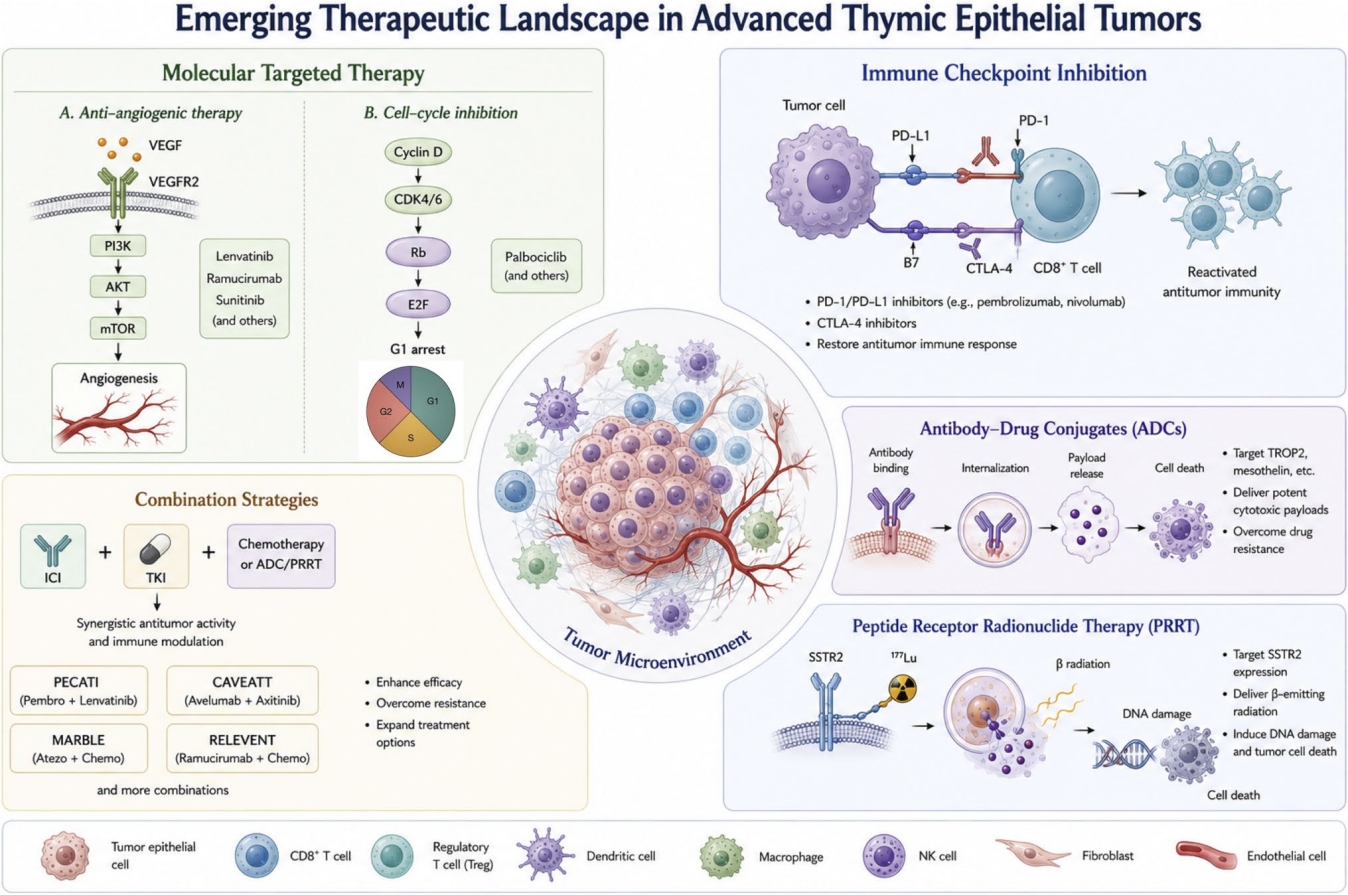
Emerging therapeutic strategies in thymic epithelial tumors

A diverse array of emerging therapeutic strategies is under active investigation in TETs (Fig. 2) [66, 67]. In patients with relapsed or refractory advanced TETs after platinum-based therapy, palbociclib monotherapy demonstrated favorable tolerability with an objective response rate of 12.5% [68]. Antibody–drug conjugates (ADCs) targeting mesothelin and trophoblast cell surface antigen-2 (Trop-2) are emerging as promising precision therapies for refractory thymic carcinoma [69, 70]. Beyond systemic agents, multimodal locoregional strategies continue to evolve; cytoreductive surgery combined with hyperthermic intrathoracic chemotherapy (HITOC) for pleural dissemination or recurrent TETs achieved encouraging outcomes with manageable morbidity (2-year PFS 82.8%; OS 100%) [71].

Multiple receptor tyrosine kinases, including epidermal growth factor receptor (EGFR), stem cell factor receptor (c-KIT), insulin-like growth factor-1 receptor (IGF-1R), and vascular endothelial growth factor (VEGF), are overexpressed in TETs, suggesting potential therapeutic roles for corresponding targeted inhibitors [72]. In the second-line setting, lenvatinib, a multitarget tyrosine kinase inhibitor against VEGFR, FGFR, RET, and c-KIT, has demonstrated antitumor activity with acceptable safety and is a treatment option for previously treated thymic carcinoma [73].

### Molecular landscape and therapeutic opportunities

Thymic epithelial tumors harbor distinct genomic profiles that fundamentally differentiate thymomas from thymic carcinomas (Table 4). GTF2I mutation is the most frequent oncogenic alteration in thymomas, occurring in up to 80% of type A and AB thymomas. In mouse models, GTF2I alterations impair thymic epithelial progenitor differentiation and lead to medullary thymic epithelial cell maturation defects and age-dependent tumor formation [74–76]. By contrast, thymic carcinomas are characterized by mutations in CDKN2A**, **TP53**,** and CDKN2B, and exhibit significantly higher tumor mutational burden than thymomas. Beyond these subtype-defining alterations, recurrent genomic variants affecting the p53, AKT, MAPK, and K-Ras signaling pathways have been identified across TETs, suggesting potential therapeutic vulnerabilities [77–83]. The YAP1-MAML2 gene fusion represents a distinctive molecular hallmark of metaplastic thymoma, a clinically indolent variant [84].

**Table 4:** Molecular alterations and targeted therapeutic opportunities in advanced thymic epithelial tumors

Molecular alteration	Primary TET subtype(s)	Frequency	Prognostic significance	Therapeutic implication	Ref
**GTF2I** mutation	Thymoma (types A/AB)	Up to 80%	Favorable	Prognostic biomarker; no targeted agent currently approved	[74–76]
**YAP1-MAML2** fusion	Metaplastic thymoma	Characteristic	Indolent	Diagnostic marker; benign clinical course	[84]
**CDKN2A/CDKN2B**, **TP53** mutations	Thymic carcinoma	Common (TP53 ~28%; CDKN2A ~12%)	Adverse	CDK4/6 inhibitors under investigation	[77, 78]
**PI3K/AKT/mTOR** pathway alterations	Metastatic thymic carcinoma	Enriched	Adverse	PI3K/mTOR inhibitors (preclinical and early clinical evidence)	[79, 80]
**KIT** mutation	Thymic carcinoma (subset)	~9–11%	Unknown	KIT inhibitors (imatinib, sunitinib); case reports of immunotherapy activity	[81]
**BRCA2** mutation	Thymoma (rare)	Rare	Unknown	PARP inhibitors (case report of clinical benefit)	[82]
**HOXD9/SALL3** hypermethylation	Thymic carcinoma	Common	Adverse	Epigenetic modulators (HDACi, DNMTi)	[83]
**ROS1**, **CDKN2A** mutations	Thymic carcinoma	Subset	Adverse	ROS1 inhibitors; CDK4/6 inhibitors	[85, 86]

ICIs immune checkpoint inhibitors, HDACi histone deacetylase inhibitors, DNMTi DNA methyltransferase inhibitors

Metastatic TETs, particularly thymic carcinomas, show genomic alterations involving TP53/CDK, EGFR/RAS, and PI3K/mTOR pathways [85, 86]. Preclinical evidence supports PI3K pathway inhibition as a potential therapeutic strategy in thymic carcinoma [87], and subsets of thymic carcinomas harboring KIT mutations with concurrent PD-L1 overexpression may be amenable to combined targeted and immunotherapeutic approaches [88]. However, our understanding of molecular alterations in thymic tumors remains incomplete, and targeted therapy has not yet become standard of care in metastatic thymoma. Nevertheless, emerging evidence suggests that BRCA2-mutant thymomas may derive clinical benefit from PARP inhibitors [89, 90].

Prognostically, GTF2I mutation consistently correlates with favorable outcomes [91], whereas hypermethylation of HOXD9 and SALL3 is associated with poor prognosis [92]. In thymic carcinoma, mutations in ROS1 and CDKN2A are significantly associated with adverse survival, with prognostic genes predominantly enriched in cell cycle regulation, chromatin remodeling/DNA methylation, PI3K/AKT/mTOR, and RTK/RAS/MAPK pathways [93].

Beyond genomic alterations, epigenetic changes offer diagnostic and therapeutic opportunities. DNA methylation-based classifiers provide a novel tool for precise TET subclassification [94], and noncoding RNA clusters and methylation changes may serve as potential biomarkers. Histone deacetylase inhibitors have demonstrated notable antitumor activity in clinical trials [95], and epigenetic regulation of miR-145-5p along with its targeting pathways plays a critical role in TET progression and treatment response [96]. Collectively, these findings position epigenetic modulators as an emerging therapeutic strategy in TETs.

### Thymic neuroendocrine tumors

Thymic neuroendocrine tumors (TNETs) are rare but aggressive malignancies with limited therapeutic options [97]. The 2021 World Health Organization (WHO) classification has refined diagnostic framework, and insulinoma-associated protein 1 (INSM-1) has emerged as a highly sensitive marker across all subtypes [98]. Molecular profiling has identified distinct genomic features, including frequent MEN1, MLL2, APC, and RB1 mutations, with MEN1-associated TNETs representing a unique transcriptomic subgroup [93, 99].

Clinically, TNETs often present at advanced stage; ectopic Cushing syndrome in up to 15% of cases portends a poor prognosis [100, 101]. Complete surgical resection remains the cornerstone of curative treatment, independently associated with improved survival; lymph node dissection is warranted given the high rate of nodal metastasis [102]. For advanced disease, platinum-etoposide chemotherapy yields an objective response rate of ~31% [103]; somatostatin analogs, everolimus, and cabozantinib represent additional options [104–106]. The combination of lenvatinib and pembrolizumab has shown encouraging activity in platinum-refractory thymic malignancies, and peptide receptor radionuclide therapy (PRRT) is under prospective evaluation in the phase III LEVEL trial [107, 108]. ICI holds promise given the “immune-inflamed” phenotype of TNETs, though responses remain variable [109]. Given the paucity of dedicated prospective data, subtype-specific clinical trials are urgently needed to establish standardized treatment paradigms for this challenging malignancy.

## Conclusion

The management of thymic epithelial tumors has become increasingly individualized, reflecting advances in surgical techniques, molecular characterization, and systemic therapies. Nevertheless, important challenges remain, particularly in defining the optimal extent of thymic resection, mitigating immune-related toxicity associated with immune checkpoint inhibitors, and translating molecular insights into clinically effective therapeutic strategies. Continued progress will depend on biomarker-driven patient selection, rational integration of multimodality treatment, and prospective collaborative studies capable of generating high-quality evidence for these rare malignancies.

## Data Availability

No datasets were generated or analyzed during the current study.
